# The impact of health education intervention on knowledge, attitude, and practice of office workers about office ergonomics: a randomized controlled trial

**DOI:** 10.1186/s12889-026-29505-4

**Published:** 2026-10-07

**Authors:** Faten Ezzelarab Younis, Sally Abdelwanees, Amira Mohamed Abdel-Monaem, Shaimaa Yaihya Abdel-Raouf

**Affiliations:** Department of Public Health and Community Medicine, Faculty of Medicine, Menoufia University, Menoufia governorate, Shebin El kom city, Egypt

**Keywords:** Ergonomics, Health education, KAP, Office workers, Predictors

## Abstract

**Background:**

Ergonomics is fundamental to improving safety and efficiency in offices. Therefore, the purpose of this study was to assess the effect of a health education intervention on office workers’ knowledge, attitude, and practice (KAP) regarding ergonomics and to determine their predictors.

**Methods:**

For this intervention study, 230 office workers were split into two equal groups. A validated KAP office ergonomics questionnaire and a health education intervention were given to the intervention group, while the control group only received the questionnaire. Three months later, both groups received a post-test questionnaire.

**Results:**

The post-test KAP scores of the intervention group were significantly higher than those of the control group (37.2 versus 30.9, 23.4 versus 28.3, and 17.0 versus 10.3, respectively, *P* < 0.05). Workers’ office ergonomics knowledge, attitude, and practice scores significantly improved following the health education intervention (*P* < 0.05). Adequate knowledge and a positive attitude were significantly predicted by being younger (adjusted OR: 7.8 and 10.7), having a high level of education (adjusted OR: 8.9 and 2.5), using a computer (adjusted OR: 7.0 and 5.0), working long hours on a computer (adjusted OR: 3.1 and 3.0), and having a short job duration (adjusted OR: 5.2 and 12.3) within the intervention group. There was a significant positive correlation between the knowledge and practice scores (*r* = 0.469, *P* < 0.05).

**Conclusions:**

The outcomes of the study demonstrated that office workers who received health education had significantly enhanced their KAP after a three-month follow-up. This highlights the importance of office workers’ ergonomics education to ensure workplace safety and improve their well-being and productivity.

**Trial registration:**

The study was retrospectively registered at the Pan African Clinical Trial Registry (PACTR) (ID: PACTR202604893125732) on 29 April 2026.

**Supplementary Information:**

The online version contains supplementary material available at https://doi.org/10.1186/s12889-026-29505-4.

## Introduction

Ergonomics is the study of matching job requirements and the work environment to the employee to maximise the quality, quantity, and efficiency of work while minimising overexertion, fatigue, and work-related musculoskeletal disorders (MSDs) [1]. According to the World Health Organization (WHO), MSDs are a major contributor to the global burden of disease, affecting 1.71 billion people worldwide [2]. Ergonomics aims to enhance comfort, safety, and productivity. It plays a critical role in reducing the risk of injury and improving user experience in both occupational and daily settings [3]. It seeks to ensure that human limitations and capabilities are considered during the design process and is applied in offices, manufacturing environments, and consumer product design [4]. Ergonomic risk factors include jobs requiring repetitive, forceful, or sustained upper-extremity use, pushing, frequent lifting, pulling heavy objects, and maintaining uncomfortable postures for extended periods [1].

Ergonomics eliminates barriers that prevent workers from performing at their best and helps prevent workplace injuries [5]. It improves workers’ quality of life and earning capacity [6]. In addition, ergonomics enhances work productivity by lowering medical expenses and lost man-hours, ultimately improving organisational performance and providing long-term benefits to society [7]. However, adherence to its principles remains poor globally, especially in low- and middle-income countries [8]. Ergonomics has been extensively studied among different occupational groups [6, 9, 10].

Health education is a fundamental component of MSD prevention and management, aiming to improve individuals’ understanding of MSDs, increase awareness of ergonomic risk factors, promote self-management, and facilitate the adoption of healthy behaviors to reduce injury risk and improve functional outcomes [11]. Contemporary clinical practice guidelines consistently recommend incorporating patient education as a core element of high-quality care for MSDs alongside other conservative interventions, reflecting the current shift toward patient-centered management that emphasizes self-management, behavioral change, and maintenance of functional activity rather than relying solely on therapeutic interventions [11, 12].

Although engineering controls, workstation modifications, and other ergonomic interventions remain essential for reducing occupational risk factors, health education represents a practical, cost-effective, and sustainable strategy that empowers workers to apply ergonomic principles in their daily activities and maintain long-term healthy work behaviors [13]. Furthermore, studies have demonstrated that health education improves adherence to workplace ergonomics principles [14]. Despite its recognized importance, evidence on the optimal educational approach and its long-term effectiveness remains limited, and structured health education interventions have been relatively underexplored in musculoskeletal research, highlighting the need for further well-designed intervention studies [11, 15].

In occupational settings, particularly among office workers, ergonomic interventions have focused on flexible workspace design, including visual display terminals and adjustable keyboards and chairs [4, 16, 17]. Office workers are susceptible to ergonomic hazards for various reasons, such as prolonged sitting, frequent computer use, and static or awkward postures during daily tasks. These working conditions increase the risk of MSDs in the neck, shoulders, back, and upper limbs, which may subsequently negatively affect workers’ health, productivity, and quality of life [17]. Ergonomic interventions among office workers include training programmes, equipment modifications, and workplace modifications that aim at reducing the incidence and severity of MSDs [17–19]. Therefore, improving office workers’ ergonomic knowledge and appropriate practices is crucial to prevent these negative outcomes. A previous study in Nigeria demonstrated that a health education intervention enhanced office workers’ knowledge, attitudes, and ergonomic practices [20]. In addition, previous studies have reported that ergonomic training programmes reduced the risk of MSDs among office workers [17–19].

To the best of our knowledge, little is known about ergonomic principles among office workers and the effects of health education programmes in Egypt. Therefore, the purpose of this study was to assess the impact of a health education intervention on office ergonomics knowledge, attitude, and practice (KAP) among office workers at the Faculty of Medicine, Menoufia University, Egypt, and to determine the predictors of favourable KAP.

## Participants and methods

### Study design, timing, and setting

This randomized controlled trial, which focused on a health education intervention, was carried out at Menoufia University’s Faculty of Medicine in Egypt between July and November of 2025. This study follows the guidelines outlined in the CONSORT 2025 checklist [21]. Supplementary file 1 contains the completed checklist.

#### Calculation of sample size

The sample size was determined using G*Power 3.1.9.2 (Universität Kiel, Germany), based on the following criteria: The study’s 0.05 alpha error and 80% power were taken into account, along with the mean difference in knowledge after health education (2.05) and the SD difference (9) from a previous study [22]. The calculated sample size was 110 participants for each group, with a group ratio of 1:1. Following the addition of 10% for dropouts, each group needed a total of 121 participants.

#### Inclusion and exclusion criteria

Full-time office workers employed at the Faculty of Medicine, Menoufia University, who performed desk-based administrative work, regardless of age or sex, were eligible to participate. A list of eligible office workers was obtained from the faculty’s human resources department. Only the office workers who might retire before three months or refused participation were excluded because this study needed three months of follow-up.

### Recruitment procedure

Eligible office workers were approached in their workplaces and invited to participate in the study. The study objectives and procedures were explained before enrollment. Participation was entirely voluntary. Those who agreed to participate provided verbal informed consent. Participants were informed that they had the right to decline participation or withdraw from the study at any time without any consequences.

### The sampling method

Although the calculated sample size was 121 participants per group, all eligible office workers at the Faculty of Medicine, Menoufia University, were invited to participate (total population sampling). After applying the inclusion and exclusion criteria, a total of 230 eligible office workers were available. Therefore, all eligible office workers were included in the study and subsequently randomized equally into the intervention and control groups.

#### Randomization

Eligible participants were assigned a unique identification number. A computer-generated randomization list was created using Microsoft Excel. Participants were then allocated in a 1:1 ratio according to their random identification numbers, with odd numbers assigned to the intervention group and even numbers assigned to the control group. Each group included 115 office workers.

### Tools

This study used a validated KAP questionnaire on office ergonomics and a health education intervention. Permission to use the predesigned questionnaire was obtained from the corresponding author of the original study [23]. The questionnaire had previously undergone content validation, and its internal consistency was acceptable to good, with Cronbach’s alpha values of 0.67, 0.78, and 0.81 for the knowledge, attitude, and practice sections, respectively [23]. The questionnaire was administered through face-to-face interviews by trained data collectors at baseline (before the intervention) and again three months after the intervention. The questionnaire was administered in Arabic, which was the native language of all participants.

This questionnaire contained three parts as well as sociodemographic and occupational information. The first component consisted of ten questions about knowledge of office ergonomics covering the concept of ergonomics, workplace application, symptoms and risk factors of MSDs, proper workstation setup, correct body posture, position changes during work, and preventive ergonomic measures. On a Likert scale of one to four, the answers to these questions were 1 for poor, 2 for fair, 3 for good, and 4 for very good. Ten elements were added up to create the total score, which varied from 10 to 40. A higher score indicated more knowledge.

The second section covered office ergonomics attitudes, including sixteen items addressing appropriate workstation arrangement, regular work breaks, ergonomic risk factors, workplace policies, and occupational health measures for preventing MSDs. The five choices for each item were scaled from one to five: 1 for strongly agree, 2 for agree, 3 for neutral, 4 for disagree, and 5 for strongly disagree. The total score from the summation of sixteen questions’ answers ranged from 16 to 80. The higher total score indicates a lower attitude.

The third component included five items on participants’ ergonomics practices related to daily work habits such as twisting, bending, and repetitive tasks. Each item had a Likert range from one to four: 1 for always, 2 for sometimes, 3 for rarely, and 4 for never. The summation of the answers to five questions led to the total practice score, which ranged from 5 to 20. The higher total score was for better practice.

**Fig. 1:**
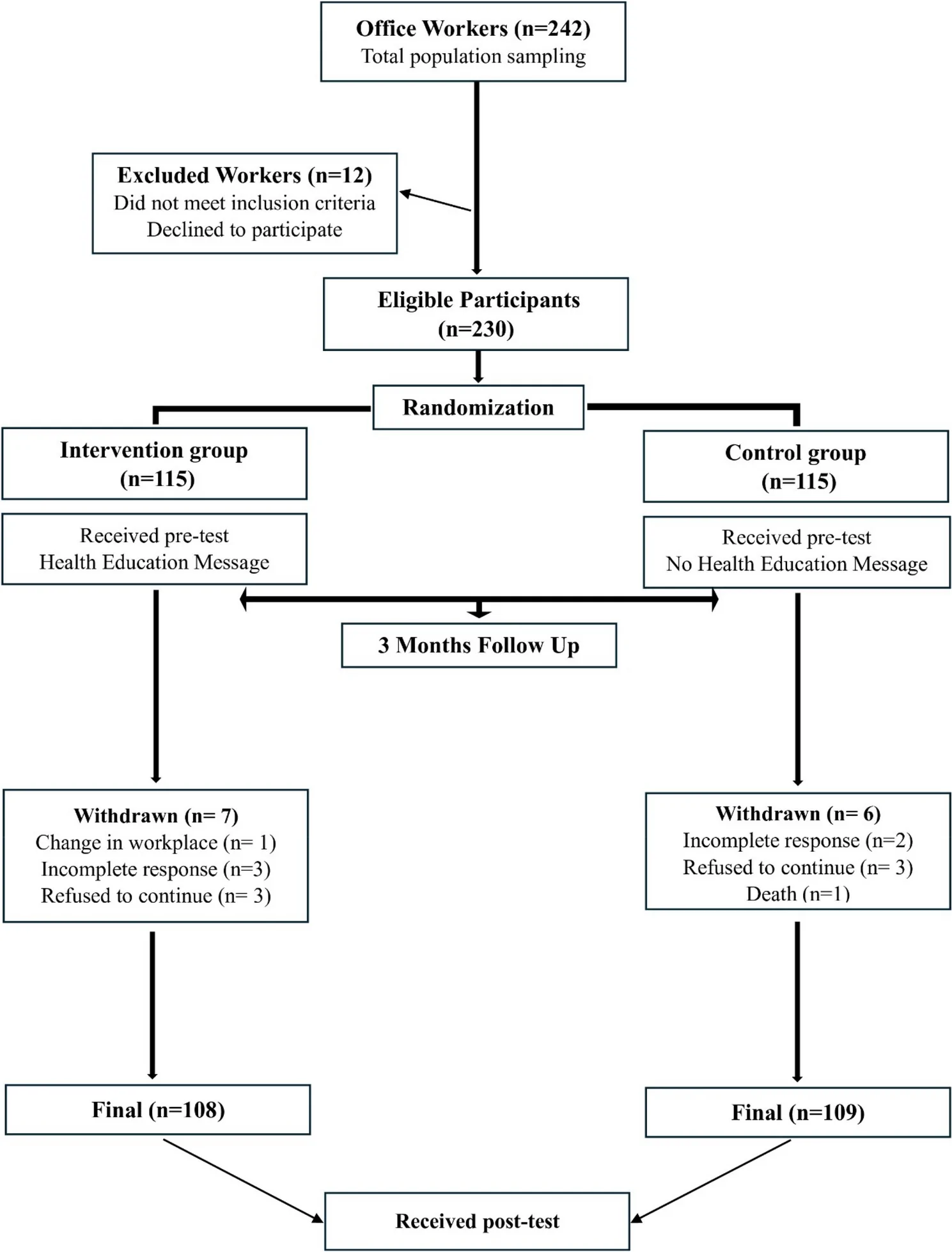
Flowchart of the included office workers

The intervention process has three phases that are illustrated in Fig. 1. During the pre-intervention phase, the pre-designed KAP questionnaire was given to all participating office workers of both groups (*n* = 230) to complete as a pre-test. Every group was in a separate auditorium at Menoufia University’s Faculty of Medicine. During the intervention phase, only the intervention group received a one-hour health education session immediately after completing the pre-test questionnaire. The session was delivered by the research team using a PowerPoint presentation with the aid of a computer and projector. The educational materials were developed by the authors based on current ergonomic guidelines and relevant literature. The educational content covered the concept and importance of office ergonomics, risk factors and symptoms of MSDs, correct workstation setup, proper sitting posture, monitor, keyboard, and chair adjustment, the importance of regular breaks and position changes, stretching exercises, and recommended ergonomic practices for preventing MSDs. Participants were encouraged to ask questions and discuss practical applications throughout the session.

Three months after the intervention, the post-intervention phase was conducted. Both groups underwent another evaluation. For various reasons, as seen in Fig. 1, thirteen participants withdrew. One hundred and eight office workers from the intervention group and 109 participants from the control group answered the same KAP questionnaire as a post-test.

### Blinding

It was not feasible in this trial because the intervention consisted of a health education session delivered only to the intervention group. Therefore, participants were aware of whether they received the educational session, and the researchers delivering the intervention were also aware of group allocation.

### Outcome measures

The primary outcome was how the KAP scores changed between and within each group after three months of health education intervention. The secondary outcomes examined which occupational and sociodemographic characteristics predicted the improvement in KAP scores, such as age, education level, working hours, and length of employment.

### Statistical analysis

Software for statistics (SPSS version 20) was used to gather and analyse the data. The subjective variables between the intervention and control groups were compared using the chi-square test or Fisher’s exact test, where appropriate. In contrast, the quantitative data between the two groups was compared using the students’ t-test or Mann-Whitney test, depending on normality. The total score for each of knowledge, attitude, and practice was calculated for both pre- and post-tests. The student t-test was used to compare the differences in each score between the two groups under study, and the paired t-test was used to evaluate the changes within each group. The correlation between scores of the practice and knowledge was examined using Pearson correlation. The median of each score of KAP was used to split into two categories. The values of KAP scores equal to or above the median were categorized as adequate knowledge, positive attitude, and favorable practice, respectively, while those below the median were classified as inadequate knowledge, negative attitude, and unfavorable practice, respectively. In the binary logistic regression, these categories served as the dependent variables. Both sociodemographic and occupational variables were entered simultaneously into the regression model using the Enter method to identify independent predictors of favorable KAP. Adjusted odds ratios (aORs) with 95% confidence intervals (CIs) were reported. Variables that were not statistically significant were retained in the model to adjust for potential confounding. A p-value below 0.05 was the level of significance.

## Results

Two hundred and thirty office workers were recruited for this intervention study, and they were randomly allocated to the intervention group (*n* = 115) and the control group (*n* = 115). Baseline characteristics of all enrolled participants are presented in Table 1. The participants’ ages ranged from 26 to 59, with a mean ± SD of 40.4 ± 9.3. There were 58.7% female office workers. 89.6% of the participants were married. Approximately half of them had a university or master’s degree, and 51.3% of them were residents of urban areas. 87.8% of them used computers, and the median number of hours spent using a computer was 17.5, with an interquartile range of 7–24. Twelve years was the median length of their employment, and thirty-five hours was the median of their working hours per week. Sociodemographic and occupational factors did not significantly differ between the two groups (*P* > 0.05) (Table 1).

**Table 1:** Sociodemographic and occupational characteristics among the studied groups

Parameter	Total participants (*n* = 230)	Intervention group (*n* = 115)	Control group (*n* = 115)	Test of significant	*P* value
Sex					
Male	95 (41.3%)	44 (38.3%)	51 (44.3%)		
Female	135 (58.7%)	71 (61.7%)	64 (55.7%)	χ^2^ = 0.87	0.34
Age (year)					
Mean ± SD	40.4 ± 9.3	40.5 ± 8.7	40.3 ± 10.0		
Range	26–59	27–59	26–59	t = 0.16	0.86
Residence					
Urban	118 (51.3%)	55 (47.8%)	63 (54.8%)		
Rural	112 (48.7%)	60 (52.2%)	52 (45.2%)	χ^2^ = 1.11	0.29
Marital status					
Married	206 (89.6%)	104 (90.4%)	102 (88.7%)		
Unmarried	24 (10.4%)	11 (9.6%)	13 (11.3%)	χ^2^ = 0.18	0.66
Level of education					
Secondary/below	114 (49.6%)	59 (51.3%)	55 (47.8%)		
University/master	116 (50.4%)	56 (48.7%)	60 (52.2%)	χ^2^ = 0.27	0.59
Use of computer					
Yes	202 (87.8%)	104 (90.4%)	98 (85.2%)		
No	28 (12.2%)	11 (9.6%)	17 (14.8%)	χ^2^ = 1.46	0.22
Computer working hours per week					
Median (IQR)	17.5 (7–24)	17.5 (3.5–24)	17.5 (10–24)	U = 1.56	0.11
Years of employment					
Median (IQR)	12 (8–21)	13 (8–21)	11 (7–23)	U = 0.44	0.65
Number of working hours per week					
Median (IQR)	35 (30–40)	35 (30–40)	40 (30–40)	U = 0.05	0.95

*SD* standard deviation, *IQR* interquartile range, *t* Student’s t-test, χ^*2*^ chi square test, *U* Mann Whitney test

During the three-month follow-up, 13 participants withdrew from the study. A change in workplace (*n* = 1), incomplete responses (*n* = 3), and refusal to continue (*n* = 3) were the reasons for withdrawals in the intervention group. Withdrawals in the control group were caused by incomplete responses (*n* = 2), refusal to continue (*n* = 3), and death (*n* = 1) (Fig. 1). Therefore, the post-intervention analysis included 217 participants (108 in the intervention group and 109 in the control group), as shown in Table 2.

**Table 2:** The pre- and post-knowledge, attitude, and practice scores among the studied groups

Parameter		Intervention group (*n* = 115)	Control group (*n* = 115)	Student’s t-test	P value
		Mean ± SD	Mean ± SD		
Pre-test	Knowledge score	30.6 ± 4.1	30.5 ± 3.5	0.13	0.89
	Attitude score	28.6 ± 6.2	27.9 ± 7.0	0.77	0.44
	Practice score	10.4 ± 1.9	10.1 ± 2.4	1.36	0.17

Parameter		Intervention group (*n* = 115)	Control group (*n* = 115)	Student’s t-test	P value
		Mean ± SD	Mean ± SD		
Post-test	Knowledge score	37.2 ± 1.6	30.9 ± 3.3	17.87	< 0.001*
	Attitude score	23.4 ± 3.9	28.2 ± 6.7	6.47	< 0.001*
	Practice score	17.0 ± 1.9	10.3 ± 2.6	20.72	< 0.001*

*Significant difference, *SD* standard deviation

There was no significant difference between the intervention and control groups when comparing each total KAP score prior to the health education intervention as a pretest (knowledge score: 30.6 ± 4.1 versus 30.5 ± 3.5; attitude score: 28.6 ± 6.2 versus 27.9 ± 7.0; and practice score: 10.4 ± 1.9 versus 10.1 ± 2.4, *P* > 0.05). However, after the intervention group received health education and underwent three months of follow-up, there was a significant improvement in the KAP scores between both groups (knowledge score: 37.2 ± 1.6 versus 30.9 ± 3.3; attitude score: 23.4 ± 3.9 versus 28.3 ± 6.7; and practice score: 17.0 ± 1.9 versus 10.3 ± 2.6, *P* < 0.05) (Table 2).

**Fig. 2:**
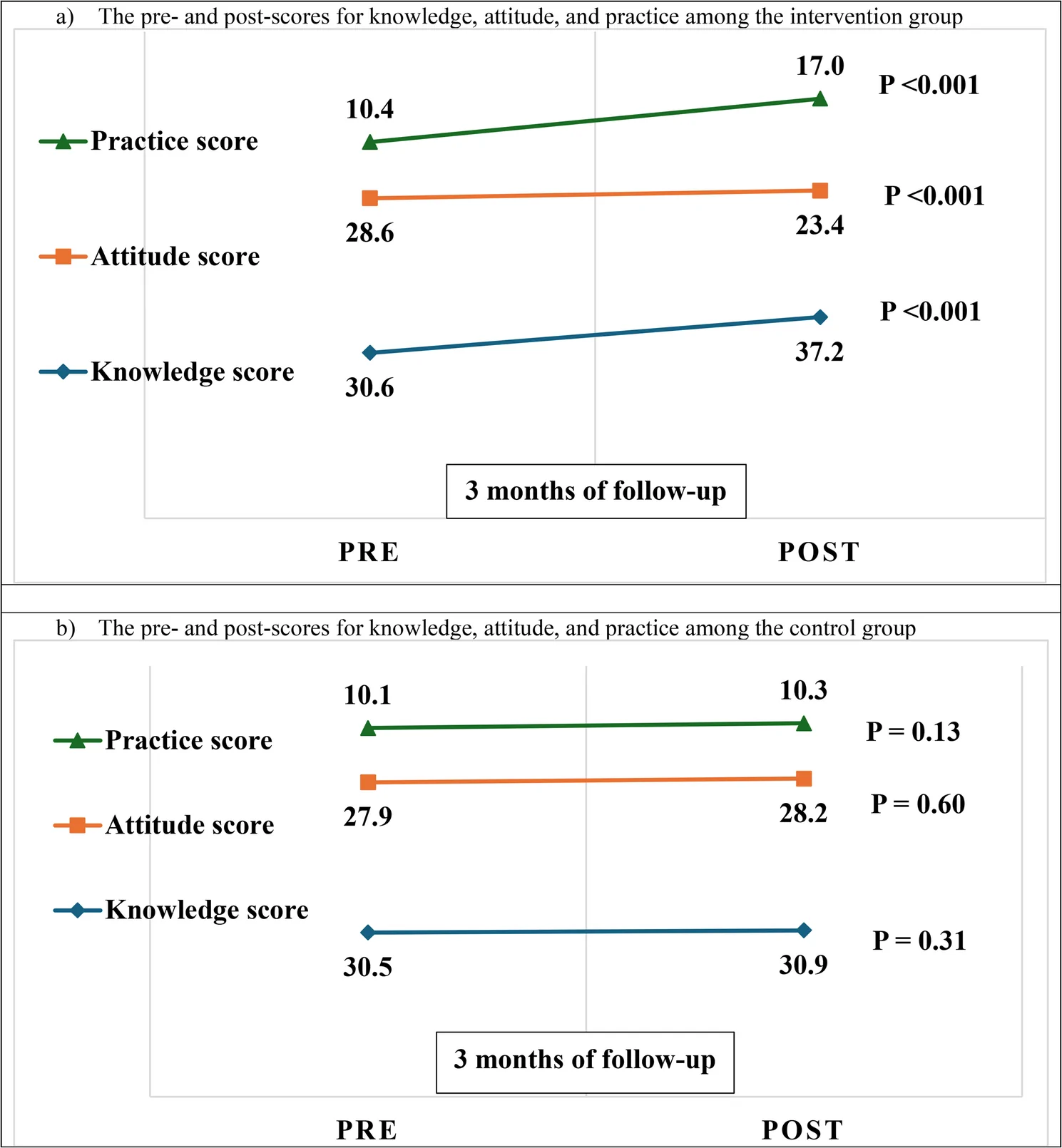
The pre- and post-scores for knowledge, attitude, and practice within each group

After three months of follow-up, the control group’s KAP scores did not significantly alter between the pre- and post-test (knowledge score: 30.5 ± 3.5 versus 30.9 ± 3.3; attitude score: 27.9 ± 7.0 versus 28.3 ± 6.7; and practice score: 10.1 ± 2.4 versus 10.3 ± 2.6, *P* > 0.05). However, within the intervention group, KAP scores changed significantly between pre- and post-test (knowledge score: 30.6 ± 4.1 versus 37.2 ± 1.6; attitude score: 28.6 ± 6.2 versus 23.4 ± 3.9; and practice score: 10.4 ± 1.9 versus 17.0 ± 1.9, *P* < 0.05) (Fig. 2).

**Fig. 3:**
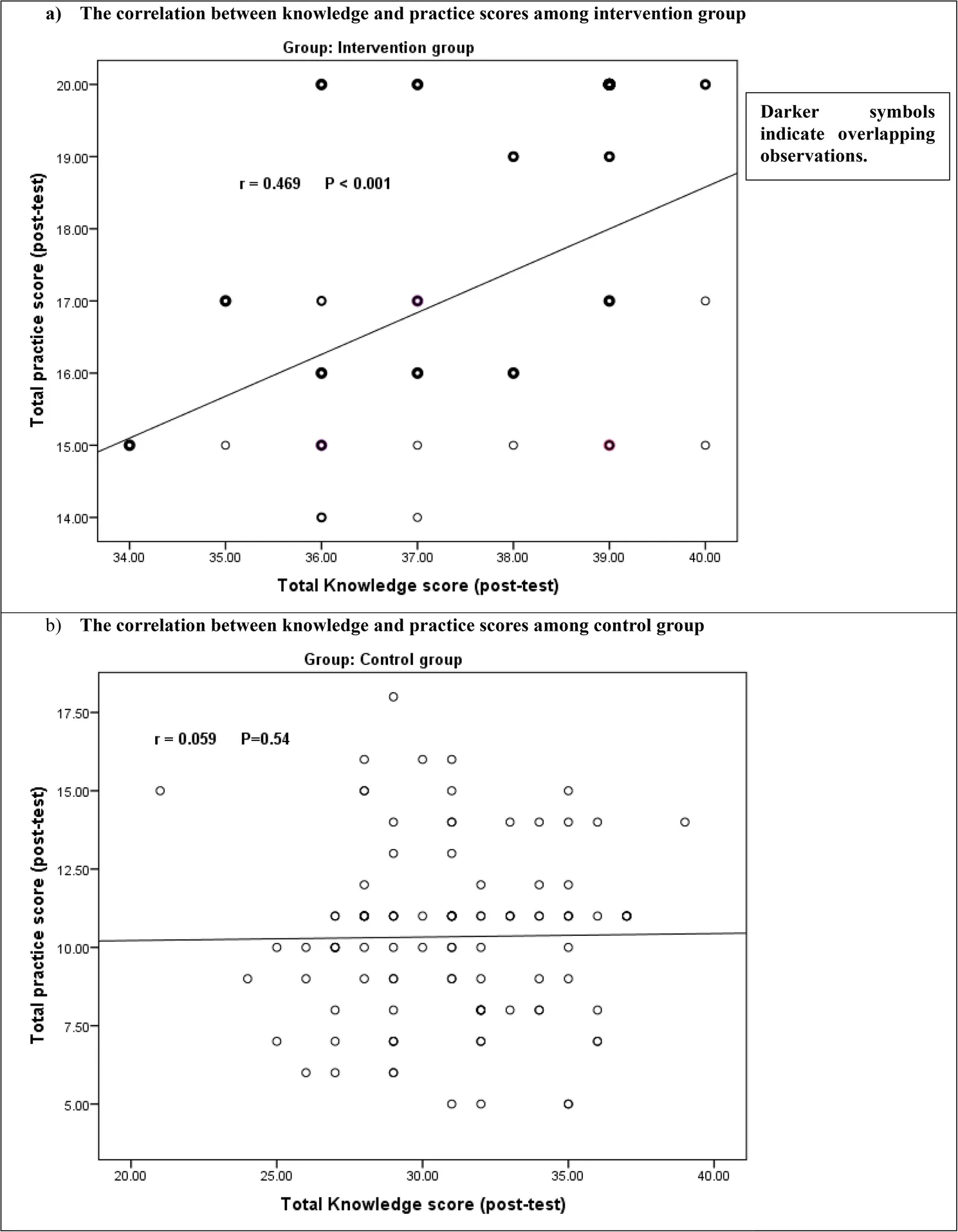
The correlation between post-test knowledge and practice scores among the studied groups

The knowledge score was positively correlated with the practice score in the intervention group after three months of follow-up (*r* = 0.469, *P* < 0.05) (Fig. 3a). However, the control group did not show any significant correlation (*r* = 0.059, *P* > 0.05) (Fig. 3b).

After 3 months of follow-up within the intervention group, the percentage of adequate knowledge, positive attitude, and favourable practice was 62.9% (68/108), 57.4% (62/108), and 68.5% (74/108), respectively (Table 3).

**Table 3:** The predictors for knowledge, attitude, and practice among the intervention group

Parameter	Intervention group after three months of follow-up (*n* = 108)							
	Knowledge		Adjusted OR (95% CI)	Attitude		Practice		Adjusted OR (95% CI)
	Inadequate (*n* = 40)	Adequate (*n* = 68)		Negative (*n* = 46)	Positive (*n* = 62)	Unfavorable (*n* = 34)	Favorable (*n* = 74)	
Age (year)								
<41	8 (20.0%)	45 (66.2%)	7.8	22 (47.8%)	31 (50.0%)	5 (14.7%)	48 (64.9%)	10.7
≥41 (ref.)	32 (80.0%)	23 (33.8%)	(3.1–19.7)*	24 (52.2%)	31 (50.0%)	29 (85.3%)	26 (35.1%)	(3.7–30.9)*
Sex								
Male	13 (32.5%)	28 (41.2%)	1.4	18 (39.1%)	23 (37.1%)	13 (38.2%)	28 (37.8%)	1.1
Female (ref.)	27 (67.5%)	40 (58.8%)	(0.6–3.2)	28 (60.9%)	39 (62.9%)	21 (61.8%)	46 (62.2%)	(0.4–2.6)
Residence								
Urban	13 (32.5%)	37 (54.4%)	2.4	19 (41.3%)	31 (50.0%)	13 (38.2%)	37 (50.0%)	1.2
Rural (ref.)	27 (67.5%)	31 (45.6%)	(1.0-5.6)*	27 (58.7%)	31 (50.0%)	21 (61.8%)	37 (50.0%)	(0.5–2.9)
Marital status								
Married (ref.)	39 (97.5%)	59 (86.8%)	5.9	42 (91.3%)	56 (90.3%)	33 (97.1%)	65 (87.8%)	4.5
Unmarried	1 (2.5%)	9 (13.2%)	(0.7–48.8)	4 (8.7%)	6 (9.7%)	1 (2.9%)	9 (12.2%)	(0.5–37.6)
Level of education								
Secondary/below (ref.)	32 (80.0%)	21 (30.9%)	8.9	21 (45.7%)	32 (51.6%)	22 (64.7%)	31 (41.9%)	2.5
University/master	8 (20.0%)	47 (69.1%)	(3.5–22.6)*	25 (54.3%)	30 (48.4%)	12 (35.3%)	43 (58.1%)	(1.0-5.8)*
Use of computer								
Yes	33 (82.5%)	66 (97.1%)	7.0	44 (95.7%)	55 (88.7%)	28 (82.4%)	71 (95.9%)	5.0
No (ref.)	7 (17.5%)	2 (2.9%)	(1.3–35.5)*	2 (4.3%)	7 (11.3%)	6 (17.6%)	3 (4.1%)	(1.1–21.6)*
Computer working hours per week								
< 20 (ref.)	29 (72.5%)	31 (45.6%)	3.1	21 (45.7%)	39 (62.9%)	25 (73.5%)	35 (47.3%)	3.0
≥ 20	11 (27.5%)	37 (54.4%)	(1.3–7.3)*	25 (54.3%)	25 (37.1%)	9 (26.5%)	39 (52.7%)	(1.2–7.5)*
Years of employment								
< 11	9 (22.5%)	41 (60.3%)	5.2	22 (47.8%)	28 (45.2%)	4 (11.8%)	46 (62.2%)	12.3
≥ 11 (ref.)	31 (77.5%)	27 (39.7%)	(2.1–12.6)*	24 (52.2%)	24 (54.8%)	30 (88.2%)	28 (37.8%)	(3.9–36.6)*

*Significant difference, *OR* odds ratio, *CI* confidence interval, *Ref* reference

By logistic regression analysis within the intervention group, there were significant predictors for adequate knowledge and favourable practice: being younger (adjusted OR: 7.8 with 95% CI: 3.1–19.7 and adjusted OR: 10.7 with 95% CI: 3.7–30.9, *P* < 0.05), having a high level of education (adjusted OR: 8.9 with 95% CI: 3.5–22.6 and adjusted OR: 2.5 with 95% CI: 1.0-5.8, *P* < 0.05), using a computer (adjusted OR: 7.0 with 95% CI: 1.3–35.5 and adjusted OR: 5.0 with 95% CI: 1.1–21.6, *P* < 0.05), spending long hours working on a computer (adjusted OR: 3.1 with 95% CI: 1.3–7.3 and adjusted OR: 3.0 with 95% CI: 1.2–7.5, *P* < 0.05), and having a short work duration (adjusted OR: 5.2 with 95% CI: 2.1–12.6 and adjusted OR: 12.3 with 95% CI: 3.9–36.6, *P* < 0.05). Also, residing in an urban area was a significant predictor for adequate knowledge (adjusted OR: 2.4 with 95% CI: 1.0-5.6, *P* < 0.05) (Table 3). Binary logistic regression analysis was also performed for attitude. However, none of the variables were significantly associated with a positive attitude in the adjusted model; therefore, the adjusted ORs are not presented in Table 3.

## Discussion

The ergonomic programs have beneficial effects on the office workers’ health, job satisfaction, and productivity. Improved ergonomics knowledge and skills reduce the risk of workplace injuries [24]. This intervention study involved two equal groups of office workers, the intervention and control groups, to assess the changes in their KAP after the health education and identify potential predicting factors. The present intervention consisted of a one-hour interactive health education session on office ergonomics covering ergonomic principles and preventive practices for MSDs.

The pre-test for KAP about office ergonomics in this study showed no significant difference between the intervention and control groups, but the intervention group’s post-test scores three months after health education were significantly greater than those of the controls. This is consistent with earlier research [25, 26].

Within the intervention group, this experiment demonstrated a significant improvement in the KAP scores after three months of follow-up post health education. In contrast, the control group of this investigation did not experience this improvement in post-KAP scores. The influence of health education in the intervention group could be to blame for this. It was consistent with a trial conducted in Nigeria, where a one-day health education programme consisting of interactive lectures on workplace safety improved the knowledge, attitudes, and workplace safety practices of 247 administrative staff at one and three months of follow-up [20]. A prior Iranian study used lectures and posture training over eight weekly sessions and demonstrated significant improvements in 180 office workers’ post-intervention awareness, attitude, and practice [22]. Office workers’ behaviour enhanced following a training intervention based on the Health Promotion Model, according to another study conducted in Iran [26]. In a study conducted in India, nursing office workers’ knowledge and attitude scores rose significantly following training, while their practice scores enhanced somewhat [27]. According to research carried out in the United States, office workers’ post-test ergonomics knowledge scores increased by almost 30%, and office workers who received ergonomics training supplemented with printed guidelines and follow-up email reminders were more qualified to modify their workspace and make more efficient and ergonomic use of its facilities [28]. Another study among multinational office workers found that effective workplace ergonomic training delivered through either a 40-minute PowerPoint lecture or a 10-minute live workstation demonstration resulted in improved knowledge, changed behaviour, and more skills [29].

The effectiveness of the intervention may also have been influenced by the workplace culture, including management support and organizational commitment to ergonomics, which could affect the adoption and sustainability of ergonomic practices in different settings.

In this study, a statistically significant but moderate positive correlation (*r* = 0.469) was observed between the increase in knowledge scores and the improvement in practice scores among participants who received health education. Although this finding suggests that increased knowledge may contribute to improved ergonomic practices that prevent repetitive strain injuries and result in enhancing office workers’ well-being and workplace productivity, the magnitude of the correlation indicates that other factors are also likely to influence practice. In 2023, a review for improving workplace health and ergonomics also documents these implications [4].

Improving office workers’ KAP toward ergonomics had a beneficial impact, which is in line with earlier research proving that ergonomic intervention programs reduced occupational injuries, such as musculoskeletal problems [17–19, 28, 30–32].

Within the intervention group of this study, favourable knowledge and practice were significantly predicted by some sociodemographic and occupational characteristics, such as younger age, shorter work duration, high levels of education, and prolonged computer use. It is compatible with a previous Nigerian study that discovered that prolonged sitting or extended computer use was a major predictor of KAP regarding workplace safety [20].

These findings could be attributed to the fact that younger and more educated office workers prefer high health literacy, which increases their receptivity to and use of ergonomics following a health education intervention. Also, the occupational risk of extended sitting and prolonged computer use among office workers serves as a potent drive to improve their understanding and practices to avoid workplace injuries.

Although the attitude scores significantly improved following the intervention, no occupational or sociodemographic indicators were found to be associated with this improvement. This may be attributed to the complex nature of the attitudes of the participants because there are different factors affecting attitudes, such as beliefs, values, and motivation, rather than sociodemographic or occupational traits.

### Strengths and limitations

This study’s design provided evidence for assessing potential changes in office workers’ KAP following health education. However, the study had some limitations. The relatively small sample size and the three-month follow-up period limited the assessment of the long-term sustainability of the intervention effects. Therefore, the findings should be interpreted with caution regarding their long-term applicability. In addition, because this study was conducted at a single employment site, the findings may not be fully generalizable to workplaces with different organizational cultures, resources, or occupational health policies.

## Conclusion

This experiment revealed that office workers who received health education showed significant improvements in their KAP after a three-month follow-up. The findings also identified the socioeconomic and occupational factors that predict KAP among the office workers. These findings highlight the importance of providing office workers with ergonomics training to improve workplace safety and enhance their productivity and well-being. Further research on various training programs and educational interventions involving large numbers of office workers is needed to evaluate the long-term impact of these initiatives. Risk groups, such as older and less educated office workers, should be targeted in future intervention research. Studies with longer follow-up periods (e.g., six months or one year) are needed to determine the sustainability of these improvements and to identify the optimal frequency of refresher training.

## Practical and public health implications

The findings of this trial suggest that incorporating health education on office ergonomics into workplace health promotion programs can effectively improve office workers’ knowledge, attitudes, and ergonomic practices. This may improve workers’ well-being, productivity, and quality of life while lowering absenteeism and the associated economic burden. Organizations should consider providing regular ergonomics training sessions, promoting appropriate workstation adjustments, encouraging periodic breaks and stretching exercises, and integrating ergonomic education into occupational health and safety policies. Special attention should be directed toward workers at higher risk, such as older workers and those with lower educational levels, to maximise the effectiveness of ergonomic interventions and reduce the risk of work-related MSDs.

## Supplementary Information

Supplementary Material 1.

## Data Availability

Data will be available on reasonable request.

## References

[1] Edwards C, Fortingo N, Franklin E. Ergonomics. In: StatPearls. Treasure Island (FL): StatPearls Publishing; 2025 Jan. Last accessed on November 2025. Available online: https://www.ncbi.nlm.nih.gov/books/NBK580551/

[2] World Health Organization. Musculoskeletal conditions. 2021. Last accessed on November 2025. Available online: https://www.who.int/news-room/fact-sheets/detail/musculoskeletal-conditions

[3] Suparti E, Wahyudi AT, Fitrianingsih A. Total ergonomics approach to analyze work system and propose improvements for increasing worker productivity. OPSI. 2023;16(2):174–87. 10.31315/opsi.v16i2.9009.

[4] Adiga U. Enhancing occupational health and ergonomics for optimal workplace well-being: a review. Int J Chem Biochem Sci. 2023;24(4):157–64. 10.56025/IJARESM.2023.11823836.

[5] Uche CM, Fanny CO. Educational ergonomics in higher education institutions in Nigeria. Makerere J High Educ. 2015;7(2):133–46. 10.4314/majohe.v7i2.9.

[6] Vural F, Sutsunbuloglu E. Ergonomics: an important factor in the operating room. J Perioper Pract. 2016;26(7–8):174–8. 10.1177/1750458916026007-804. 29328758

[7] Ünlü NS, Çavdar HR, Aydoğdu A, Kılıç A. Ergonomic improvements in service and manufacturing sectors. J Appl Eng Agric Sci. 2024;2(2):21–36. Available online:. https://dergipark.org.tr/en/pub/umtd/issue/88738/1597403.

[8] Koirala R, Nepal A. A literature review on ergonomics, ergonomics practices, and employee performance. QJMSS. 2022;4(2). 10.3126/qjmss.v4i2.50322.

[9] Gangopadhyay S. Occupational ergonomics: a special domain for the benefit of workers’ health. Indian J Occup Environ Med. 2022;26(3):135–9. 10.4103/ijoem.ijoem_209_22. PMC9674068 36408436

[10] Mishra S, Bhagat D, Borah S. Ergonomic studies on occupational health of women workers involved in agricultural industries: a systematic review. Res World Agric Econ. 2024;5(4):110–27. 10.36956/rwae.v5i4.1207.

[11] Kongsted A, Larsen AC, Gregersen MHM, et al. Patient education for chronic musculoskeletal pain: a scoping review of recommendations, effectiveness, and educational content. Chiropr Man Th. 2026;34(1):3. 10.1186/s12998-025-00614-y. PMC12865995 41484990

[12] Lin I, Wiles L, Waller R, et al. What does best practice care for musculoskeletal pain look like? Eleven consistent recommendations from high-quality clinical practice guidelines: systematic review. Br J Sports Med. 2020;54:79–86. 10.1136/bjsports-2018-099878 30826805

[13] Adamčák Š, Marko M, Bartík P, Kľocová Adamčáková Z. Short- and long-term effects of musculoskeletal health education: evidence from the performing arts students. Front Sports Act Living. 2025;7:1637680. 10.3389/fspor.2025.1637680. PMC12352326 40822739

[14] Nwosu S, Atulomah N, Nwankwo V, Jamiu S. Influence of educational-intervention program on adherence to workplace ergonomic principles among catering staff of selected universities in Ogun State, Nigeria. Babcock Univ Med J. 2024;7(1):88–95. 10.38029/babcockuniv.med.j.v7i1.193.

[15] Koehn CL, Esdaile JM. Patient education and self-management of musculoskeletal diseases. Best Pract Res Clin Rheumatol. 2008;22(3):395–405. 10.1016/j.berh.2008.02.006. 18519095

[16] Aarås A, Horgen G, Ro O. Work with the visual display unit: health consequences. Int J Hum Comput Interact. 2000;12(1):107–34. 10.1207/S15327590IJHC1201_5.

[17] Mahmud N, Kenny DT, Md Zein R, Hassan SN. Ergonomic training reduces musculoskeletal disorders among office workers: results from the 6-month follow-up. Malays J Med Sci. 2011;18(2):16–26. PMC3216214 22135582

[18] Hasani MH, Hoe VC, Aghamohammadi N, Chinna K. The role of active ergonomic training intervention on upper limb musculoskeletal pain and discomfort: a cluster randomized controlled trial. Int J Ind Ergon. 2022;88:103275. 10.1016/j.ergon.2022.103275.

[19] Sohrabi MS, Babamiri M. Effectiveness of an ergonomics training program on musculoskeletal disorders, job stress, quality of work-life and productivity in office workers: a quasi-randomized control trial study. Int J Occup Saf Ergon. 2022;28(3):1664–71. 10.1080/10803548.2021.1918930. 33870873

[20] Odu JO, Hamedon TR, Mahmud A, Baharudin R. Effectiveness of health education module on work safety culture in improving knowledge, attitudes and practices: a cluster randomized controlled trial among public sector administrative workers in Nigeria. Med J Malaysia. 2023;78(3):308–17. 37271840

[21] Hopewell S, Chan AW, Collins GS, Hróbjartsson A, Moher D, Schulz KF, et al. CONSORT 2025 Statement: updated guideline for reporting randomised trials. BMJ. 2025;388:e081123. 10.1136/bmj-2024-081123. PMC11995449 40228833

[22] Pourhaji F, Naserinia SJ, Pourhaji F, Pourhaji R, Ranjbar H, Delshad MH. Educational ergonomic intervention and work-related musculoskeletal disorders among office workers in Tehran. Iran. Int J Musculoskelet Pain Prev. 2016;1(2):61–7.

[23] AlHazim SS, Al-Otaibi ST, Herzallah NH. Knowledge, attitudes, and practices regarding ergonomic hazards among healthcare workers in a Saudi government hospital. J Multidiscip Healthc. 2022;15:1771–8. 10.2147/JMDH.S371361. PMC9420414 36042943

[24] Holt KL. Ergonomic interventions in office environments: impact on employee productivity and health. Formosa J Appl Sci. 2025;4(9):3233–46. 10.55927/fjas.v4i9.370.

[25] Sanaeinasab H, Saffari M, Valipour F, Alipour HR, Sepandi M, Al Zaben F, Koenig HG. The effectiveness of a model-based health education intervention to improve ergonomic posture in office computer workers: a randomized controlled trial. Int Arch Occup Environ Health. 2018;91(8):951–62. 10.1007/s00420-018-1336-1. 29987442

[26] Delshad MH, Tavafian SS, Kazemnejad A. Educational intervention for promoting stretching exercise behavior among a sample of Iranian office employees: applying the Health Promotion Model. J Pain Res. 2019;12:733–42. 10.2147/JPR.S183410. PMC6391131 30863146

[27] Chindhalore CA, Dakhale GN, Gupta AV. Does educational intervention change knowledge, attitude, and practice regarding pharmacovigilance among nursing officers in Central India? An interventional study. J Integr Nurs. 2022;4(3):114–9. 10.4103/jin.jin_45_22.

[28] Robertson MM, O’Neill MJ. Reducing musculoskeletal discomfort: effects of an office ergonomics workplace and training intervention. Int J Occup Saf Ergon. 2003;9(4):491–502. 10.1080/10803548.2003. 14675521

[29] Madhwani KP, Nag PK. Effective office ergonomics awareness: experiences from global corporates. Indian J Occup Environ Med. 2017;21(2):77–83. 10.4103/ijoem.IJOEM_151_17. PMC5844133 29540968

[30] Robertson MM, Huang YH, O’Neill MJ, Schleifer LM. Flexible workspace design and ergonomics training: impacts on the psychosocial work environment, musculoskeletal health, and work effectiveness among knowledge workers. Appl Ergon. 2008;39(4):482–94. 10.1016/j.apergo.2008.02.022. 18462704

[31] Robertson M, Amick BC, DeRango K, Rooney T, Bazzani L, Harrist R, et al. The effects of an office ergonomics training and chair intervention on worker knowledge, behavior and musculoskeletal risk. Appl Ergon. 2009;40(1):124–35. 10.1016/j.apergo.2007.12.009. 18336791

[32] Madhwani KP, Nag PK. Web-based KAP intervention on office ergonomics: a unique technique for prevention of musculoskeletal discomfort in global corporate offices. Indian J Occup Environ Med. 2017;21(1):18–22. 10.4103/ijoem.IJOEM_145_17. PMC5763837 29391743

