# Supplementary material for: The impact of health education intervention on knowledge, attitude, and practice of office workers about office ergonomics: a randomized controlled trial
Source: BMC Public Health. 2026 Oct 7;26:2847. doi: 10.1186/s12889-026-29505-4 (PMC13644184; doi:10.1186/s12889-026-29505-4)
Supplement: Supplementary file 1 [file 12889_2026_29505_MOESM1_ESM.docx]

CONSORT 2025 checklist

| **Section/topic** | **No** | **Item description** | **Reported on Page Number/Line Number** | **Reported on Section/Paragraph** |
| --- | --- | --- | --- | --- |
| **Title and abstract** | | | | |
| Title and structured abstract | 1a | Identification as a randomised trial | Page 1/Lines 1-2 | Title |
|  | 1b | Structured summary of the trial design, methods, results, and conclusions | Page 2/Lines 34-52 | abstract |
| **Open science** | | | | |
| Trial registration | 2 | Name of trial registry, identifying number (with URL) and date of registration | Page 2/Lines 54-55 | Below Abstract |
| Protocol and statistical analysis plan | 3 | Where the trial protocol and statistical analysis plan can be accessed | Not available due to retrospective registration | Not available |
| Data sharing | 4 | Where and how the individual de-identified participant data (including data dictionary), statistical code and any other materials can be accessed | Page 9/Lines 252-253 | Availability of data and materials |
| Funding and conflicts of interest | 5a | Sources of funding and other support (eg, supply of drugs), and role of funders in the design, conduct, analysis and reporting of the trial | Page 9/Lines 248-249 | Funding |
|  | 5b | Financial and other conflicts of interest of the manuscript authors | Page 9/Lines 246-247 | Competing interests |
| **Introduction** | | | | |
| Background and rationale | 6 | Scientific background and rationale | Page 3/Lines 64-90 | Introduction |
| Objectives | 7 | Specific objectives related to benefits and harms | Page 3/Lines 90-93 | Introduction |
| **Methods** | | | | |
| Patient and public involvement | 8 | Details of patient or public involvement in the design, conduct and reporting of the trial | Page 4/Lines 104-107 | Methods/The sampling method |
| Trial design | 9 | Description of trial design including type of trial (eg, parallel group, crossover), allocation ratio, and framework (eg, superiority, equivalence, non-inferiority, exploratory) | Page 4 | Methods |
| Changes to trial protocol | 10 | Important changes to the trial after it commenced including any outcomes or analyses that were not prespecified, with reason | N/A | N/A |
| Trial setting | 11 | Settings (eg, community, hospital) and locations (eg, countries, sites) where the trial was conducted | Page 4/Lines 95-96 | Methods/Study design, timing, and setting |
| Eligibility criteria | 12a | Eligibility criteria for participants | Page 4/Lines 106-108 | Methods/Inclusion and exclusion criteria |

|  | 12b | If applicable, eligibility criteria for sites and for individuals delivering the interventions (eg, surgeons, physiotherapists) | Page 4/Lines 106-108 | Methods/Inclusion and exclusion criteria |
| --- | --- | --- | --- | --- |
| Intervention and comparator | 13 | Intervention and comparator with sufficient details to allow replication. If relevant, where additional materials describing the intervention and comparator (eg, intervention manual) can be accessed | Page 4-5/Lines 127-138 | Methods/The intervention process |
| Outcomes | 14 | Prespecified primary and secondary outcomes, including the specific measurement variable (eg, systolic blood pressure), analysis metric (eg, change from baseline, final value, time to event), method of aggregation (eg, median, proportion), and time point for each outcome | Page 5/Lines 139-142 | Methods/Outcome measures |
| Harms | 15 | How harms were defined and assessed (eg, systematically, non-systematically) | N/A | N/A |
| Sample size | 16a | How sample size was determined, including all assumptions supporting the sample size calculation | Page 5/Lines 99-103 | Methods/ Calculation of sample size |
|  | 16b | Explanation of any interim analyses and stopping guidelines | N/A | N/A |
| **Randomisation**: | | | | |
| Sequence generation | 17a | Who generated the random allocation sequence and the method used | N/A | N/A |
|  | 17b | Type of randomisation and details of any restriction (eg, stratification, blocking and block size) | Page 4/Lines 109-111 | Methods/ Randomization |
| Allocation concealment mechanism | 18 | Mechanism used to implement the random allocation sequence (eg, central computer/telephone; sequentially numbered, opaque, sealed containers), describing any steps to conceal the sequence until interventions were assigned | Page 4/Line 109 | Methods/ Randomization |
| Implementation | 19 | Whether the personnel who enrolled and those who assigned participants to the interventions had access to the random allocation sequence | N/A | N/A |
| Blinding | 20a | Who was blinded after assignment to interventions (eg, participants, care providers, outcome assessors, data analysts) | N/A as no blinding | N/A |
|  | 20b | If blinded, how blinding was achieved and description of the similarity of interventions | N/A as no blinding | N/A |
| Statistical methods | 21a | Statistical methods used to compare groups for primary and secondary outcomes, including harms | Pages 5-6/Line 143-156 | Methods/ Statistical analysis |
|  | 21b | Definition of who is included in each analysis (eg, all randomised participants), and in which group | N/A | N/A |
|  | 21c | How missing data were handled in the analysis | N/A | N/A |
|  | 21d | Methods for any additional analyses (eg, subgroup and sensitivity analyses), distinguishing prespecified from post hoc | N/A | N/A |
| **Results** | | | | |
| Participant flow, including flow diagram | 22a | For each group, the numbers of participants who were randomly assigned, received intended intervention, and were analysed for the primary outcome | Page 16 | Figure 1 |
|  | 22b | For each group, losses and exclusions after randomisation, together with reasons | Page 16 | Figure 1 |
| Recruitment | 23a | Dates defining the periods of recruitment and follow-up for outcomes of benefits and harms | Page 16 | Figure 1 |

|  | 23b | If relevant, why the trial ended or was stopped | N/A | N/A |
| --- | --- | --- | --- | --- |
| Intervention and comparator delivery | 24a | Intervention and comparator as they were actually administered (eg, where appropriate, who delivered the intervention/comparator, how participants adhered, whether they were delivered as intended (fidelity)) | N/A | N/A |
|  | 24b | Concomitant care received during the trial for each group | N/A | N/A |
| Baseline data | 25 | A table showing baseline demographic and clinical characteristics for each group | Page 6/Lines 158-165  Page 13 | Results/Paragraph1  Table 1 |
| Numbers analysed, outcomes and estimation | 26 | For each primary and secondary outcome, by group:   - the number of participants included in the analysis - the number of participants with available data at the outcome time point - result for each group, and the estimated effect size and its precision (such as 95% confidence interval) - for binary outcomes, presentation of both absolute and relative effect size | Page 6-7/Lines 166-191  Page 13-18 | Results/Paragraphs 2-5  Tables and Figures |
| Harms | 27 | All harms or unintended events in each group | N/A | N/A |
| Ancillary analyses | 28 | Any other analyses performed, including subgroup and sensitivity analyses, distinguishing pre- specified from post hoc | N/A | N/A |
| **Discussion** | | | | |
| Interpretation | 29 | Interpretation consistent with results, balancing benefits and harms, and considering other relevant evidence | Pages 7-8/Lines 193-233 | Discussion |
| Limitations | 30 | Trial limitations, addressing sources of potential bias, imprecision, generalisability, and, if relevant, multiplicity of analyses | Page 8/Line 235-237 | Strengths and limitations |

Citation: Hopewell S, Chan AW, Collins GS, Hróbjartsson A, Moher D, Schulz KF, et al. CONSORT 2025 Statement: updated guideline for reporting randomised trials. BMJ. 2025; 388:e081123. <https://dx.doi.org/10.1136/bmj-2024-081123>

© 2025 Hopewell et al. This is an Open Access article distributed under the terms of the Creative Commons Attribution License [(https://creativecommons.org/licenses/by/4.0/](https://creativecommons.org/licenses/by/4.0/)), which permits unrestricted use, distribution, and reproduction in any medium, provided the original work is properly cited.

*We strongly recommend reading this statement in conjunction with the CONSORT 2025 Explanation and Elaboration and/or the CONSORT 2025 Expanded Checklist for important clarifications on all the items. We also recommend reading relevant CONSORT extensions. See [www.consort-spirit.org.](http://www.consort-spirit.org/)

***As the checklist was provided upon initial submission, the page number/line number reported may be changed due to copyediting and may not be referable in the published version. In this case, the section/paragraph may be used as an alternative reference.**
